# Characterization of the Esophageal Microbiota and Prediction of the Metabolic Pathways Involved in Esophageal Cancer

**DOI:** 10.3389/fcimb.2020.00268

**Published:** 2020-06-26

**Authors:** Donghang Li, Ruyuan He, Guoqiang Hou, Wei Ming, Tao Fan, Lei Chen, Lin Zhang, Wenyang Jiang, Wei Wang, Zilong Lu, Haojie Feng, Qing Geng

**Affiliations:** ^1^Department of Thoracic Surgery, Renmin Hospital of Wuhan University, Wuhan, China; ^2^Department of Thoracic Surgery, Yangxin County People's Hospital, Yangxin, China

**Keywords:** microbiota, esophageal squamous cell carcinoma, esophagectomy, 16S ribosomal RNA gene sequencing, fusobacteria

## Abstract

Esophageal microbiota plays important roles in esophageal cancer. Esophagectomy, as the most important therapeutic way, contributes to changes of esophageal microbiome. However, there are few studies examining the esophageal microbiome and the metabolic changes before and after esophagectomy. The present study characterized the esophageal microbiome of 17 patients with esophageal squamous cell carcinoma (ESCC), 11 patients with esophagogastric junction (EGJ) cancer, 15 patients at 9–12 months after radical esophagectomy and 16 healthy controls (HC). 16S ribosomal RNA gene sequencing was used to evaluate the microbiome and predict the metabolic pathways. Our results showed that the microbial diversity was significantly lower in ESCC, EGJ and post-ESCC groups than that in the HC group. The abundance of Fusobacteria was higher (7.01 vs. 1.12%, *P* = 0.039) and the abundance of Actinobacteria (1.61 vs. 4.04%) was lower in the ESCC group than that in the HC group. We found significant differences in the abundance of Bacteroidetes (20.45 vs. 9.86%, *P* = 0.026), Fusobacteria (7.01 vs. 1.66%, *P* = 0.030) between ESCC and post-ESCC groups. The results of microbial composition analysis and PICRUSt demonstrated significant differences between ESCC and HC groups. The β diversity and PICRUSt suggested that the microbial composition and metabolic pathways were similar to HC group after esophagectomy. The monitoring of the esophagus microbiota may be an essential method to predict the recurrence of tumor.

## Introduction

Esophageal cancer is a rapidly growing concern worldwide, with ~572,000 new cases annually and 509,000 deaths in 2018 (Bray et al., 2018). Esophageal squamous cell carcinoma (ESCC) is the most common histological subtype of esophageal cancer, particularly in areas of eastern Asia and eastern and southern Africa. Approximately 90% of esophageal cancer cases are ESCC in China, where the disease is a major public health problem (Arnold et al., 2015; Fitzmaurice et al., 2015). The incidence of adenocarcinoma of the esophagogastric junction (EGJ) also increased rapidly over the last few decades, accounting for one-third of all esophagogastric adenocarcinoma cases (Wu et al., 2009).

Esophageal cancer is highly invasive, and it has a poor prognosis, with a five-year survival rate of ~30% in China (Bray et al., 2018). The reported risk factors of esophageal cancer included drinking, smoking, ingestion of hot food, obesity, gastroesophageal reflux disease, and Barrett's esophagus (BE) (Bray et al., 2018; Ferlay et al., 2018). The gut microbiota has been shown to play an essential role in several cancers, including gastric cancer (Brawner et al., 2014), colon cancer (Castellarin et al., 2012; Yang et al., 2017), and pancreatic cancer (Riquelme et al., 2019). As an important part of the upper gastrointestinal tract, the esophageal mucosa is also colonized by microbes. Previous studies have reported that an imbalance of the microbiome may promote esophageal cancer development and progression. Some studies have shown lower microbial richness in the upper digestive tract to be associated with esophageal squamous dysplasia, regarded as a precursor lesion of ESCC (Yu et al., 2014). The *Fusobacterium nucleatum* and *Porphyromonas gingivalis* were associated with shorter survival and might contribute to aggressive tumor behavior through the activation of chemokines in ESCC patients (Bao et al., 2014; Yamamura et al., 2019). The microbiota of the oral cavity (Peters et al., 2017) and gastric cancer (Nasrollahzadeh et al., 2015) from patients with ESCC also revealed a decrease in overall oral microbial diversity and enrichment in *Clostridiales* and *Erysipelotrichales* in the gastric corpus of patients with ESCC. Microbiota dysbiosis, including the presence of *Veillonella, Prevotella, Haemophilus, Neisseria, Campylobacter*, and *Fusobacterium*, has also been reported in association with gastroesophageal reflux disease (GRED) and is hypothesized to contribute to the evolution toward BE and adenocarcinoma at the esophagogastric junction (EGJ) (Di Pilato et al., 2016). Although a few studies have suggested the effect of esophageal microbiota on ESCC and EGJ, further data that can reveal the microbial composition in ESCC and EGJ are needed.

The treatment of ESCC includes many choices, such as endoscopic treatment, oncological treatment, surgery, and chemoradiotherapy (Lagergren et al., 2017). Esophagectomy, as one of the most radical therapeutic methods of esophageal cancer, can achieve the goal of a radical cure for early-stage patients and extend overall survival time for middle- and advanced-stage patients. Gastroesophageal reflux has been a long-standing complication after esophagectomy due to the resection of cardia. Previous studies have shown that bile reflux after subtotal gastrectomy was associated with the presence of *Streptococcus* and *Veillonella* in gastric aspirates and *Escherichia, Klebsiella*, and *Clostridium* in the intestine (Tseng et al., 2016). However, there was no study reporting a microbial characterization of ESCC patients receiving esophagectomy. We plan to investigate the composition of the esophageal microbiota in healthy tissues, tumor tissues, and after esophagectomy in the present study.

## Materials and Methods

### Study Population and Sample Collection

Normal esophagus, esophageal squamous cell carcinoma (ESCC), esophagogastric junction cancer (EGJ, in accordance with the 8th Edition of the AJCC TNM Classification), and postoperative esophageal squamous cell carcinoma (post-ESCC) specimens were collected under electronic gastroscopy from January 2018 to June 2019 at Renmin Hospital, Wuhan University (Wuhan, China). Healthy controls (HC) were normal esophageal (upper, middle, and lower segments) specimens obtained from 16 healthy volunteers with no digestive symptoms and esophagogastric mucosal lesions, as confirmed by electronic gastroscopy. ESCC (*n* = 17) and EGJ (*n* = 11) specimens were harvested from 28 patients with primary tumors, and all EGJ cases were adenocarcinoma. Post-ESCC (*n* = 15) specimens were obtained from the esophageal stump (1.5 cm above the anastomosis site of the esophageal stump and stomach) at 9–12 months after radical esophagectomy without chemoradiotherapy. All patients were diagnosed by pathological examinations.

The exclusion criteria were as follows: a systemic infectious disease, other coexisting malignant tumors, preoperative neoadjuvant chemoradiotherapy, biotherapy, or a history of gastrointestinal surgery. Patients using antibiotics and microecological preparations (probiotics, prebiotics, or symbiotics) within 2 months were also excluded. Demographic data and general clinical information related to the microecological analysis were acquired, and written informed consent was obtained from each participant. The experiments were approved by the Institutional Review Board of the Renmin Hospital, Wuhan University.

### DNA Extraction, Amplification, and Sequencing

Microbial genomic DNA was extracted from normal esophagus, ESCC, EGJ, and post-ESCC specimens using the E.Z.N.A.® Soil DNA Kit (Omega Bio-Tek, Norcross, GA, USA). The quantity and purity of DNA were determined with a NanoDrop 2000 UV-Vis Spectrophotometer (Thermo Scientific, Wilmington, MA, USA). The hypervariable region V3-V4 directionally targeted by the bacterial 16S rRNA gene was amplified with primers (338F: 5′– ACTCCTACGGGAGGCAGCAG−3′; 806R: 5′–GGACTACHVGGGTWTCTAAT−3′) in a GeneAmp 9700 PCR System (Applied Biosystems, Foster City, CA, USA) (Edgar, 2013). The PCR products were purified using the AxyPrep DNA Gel Extraction Kit (Axygen Biosciences, Union City, CA, USA) and quantified using QuantiFluor™-ST (Promega, USA) according to the manufacturers' instructions. Purified amplicons were pooled in equimolar concentrations, and paired-end high-throughput sequencing was performed using a 2 × 300 kit on the Illumina MiSeq Platform (Illumina, San Diego, CA, USA). The protocol of Majorbio Bio-Pharm Technology Co, Ltd. (Shanghai, China) was followed.

### Data Processing and Statistical Analysis

Raw Fastq files were merged in FLASH software (Magoc and Salzberg, 2011) and quality-filtered with Trimmomatic (Bolger et al., 2014) using the following criteria (Schirmer et al., 2015): (i) reads were truncated at any site receiving an average quality score <20 over a 50 bps sliding window, which retained sequences with overlaps >10 bps and mismatches of no more than 2 bp, and (ii) sequence data were demultiplexed and assigned to samples based on barcodes (exact matches) and primers (two nucleotide mismatches were allowed). Reads containing ambiguous bases were removed.

Operational taxonomic units (OTUs) were clustered with a 97% sequence similarity cutoff using UPARSE (version 7.1 https://drive5.com/uparse/) (McDonald et al., 2012; Edgar, 2013) and a novel “greedy” algorithm that simultaneously performed chimera filtering and OTU clustering. The taxonomic assignment of each sequence was carried out using the Ribosomal Database Project (RDP) Classifier algorithm (http://rdp.cme.msu.edu/) against the Silva (SSU123) 16S rRNA database with a confidence threshold of 70% (Liu et al., 2018).

Statistical analysis was carried out using GraphPad Prism (version 8.0.2) and the Majorbio I-Sanger Cloud Platform (http://www.i-sanger.com). The Kruskal–Wallis test, Fisher's exact test, and paired *t*-test were used to compare demographic characteristics. Alpha diversity indexes were calculated using MOTHUR (version 1.30.1) (Schloss et al., 2009). Principal coordinates analysis (PCoA) based on the weighted and unweighted UniFrac distance and analysis of similarities (ANOSIM) was carried out to compare the global microbial composition at the operational taxonomic unit (OTU) level (Kageyama et al., 2019). Welch's *t-*test and the Wilcoxon rank-sum test were used to identify species at phylum and genus levels. The detection of discriminant bacterial species was performed using the linear discriminant analysis effect size (LEfSe) (Segata et al., 2011). The linear discriminant analysis score (LDA score) indicated the effect size of each OTU, and OTUs with an LDA score >3.0 were defined as differentially abundant OTUs (Yang et al., 2018; Kageyama et al., 2019). Phylogenetic investigation of communities by reconstruction of unobserved states (PICRUSt) (Langille et al., 2013) analysis was used to identify Kyoto Encyclopedia of Genes and Genomes (KEGG) biochemical pathways, and the results were visualized as a heatmap with the Multiple Experiment Viewer (version 4.9.0).

## Results

### Demographic Characteristics of All Individuals

The demographic characteristics of all individuals (*n* = 59) are shown in Table 1. No differences were observed in age, sex, alcohol intake, tobacco smoking, and TNM stage. The percentages of II and III patients in the EGJ, ESCC, and post-ESCC groups were 90.9, 88.2, and 86.7%, respectively. It is of note that gastroesophageal refluxing, which is the most common complication after esophagectomy, existed in 11 (73.3%) patients of the post-ESCC group but was not observed in other groups.

**Table 1:** Demographic characteristics of all individuals.

**Characteristic**	**EGJ (*n* = 11)**	**ESCC (*n* = 17)**	**HC (*n* = 16)**	***P^**1**^***	**ESCC (*n* = 17)**	**Post-ESCC (*n* = 15)**	***P^**2**^***
Sex							
Male, No. (%)	8 (72.7)	12 (70.6)	10 (62.5)	0.824	12 (70.6)	9 (60.0)	0.712
Female, No. (%)	3 (27.3)	5 (29.4)	6 (37.5)		5 (29.4)	6 (40.0)	
Age, mean ± SD,y	61.4 ± 6.2	61.2 ± 9.8	58.6 ± 9.8	0.896	61.2 ± 9.8	59.2 ± 4.3	0.344
Alcohol intake, No. (%)	3 (27.3)	5 (29.4)	3 (18.8)	0.763	5 (29.4)	4 (26.7)	>0.99
Tobacco smoking							
Never	4 (36.3)	6 (35.3)	9 (56.2)	0.76	6 (35.3)	8 (53.4)	0.216
Current	4 (36.3)	7 (41.2)	4 (25.0)		7 (41.2)	2 (13.3)	
Former	3 (27.3)	4 (23.5)	3 (18.8)		4 (23.5)	5 (33.3)	
TNM stage							
I	1	2	-	0.863	2	2	0.988
II	5	9	-		9	8	
III	5	6	-		6	5	

*EGJ, esophagogastric junction cancer; ESCC, esophageal squamous cell carcinoma; HC, healthy control; post-ESCC, postoperative esophageal squamous cell carcinoma; SD, standard deviation; P, p-value. P^1^: Fisher's Exact and Kruskal–Wallis test were used to compare demographic characteristics of the EGJ, ESCC and HC groups. P^2^: Fisher's Exact test and paired t-test were used to compare demographic characteristics of the ESCC and Post-ESCC groups*.

### Decreased Microbial Diversity and Richness in the ESCC, EGJ, and Post-ESCC Groups Compared With the HC Group

**Figure 1:**
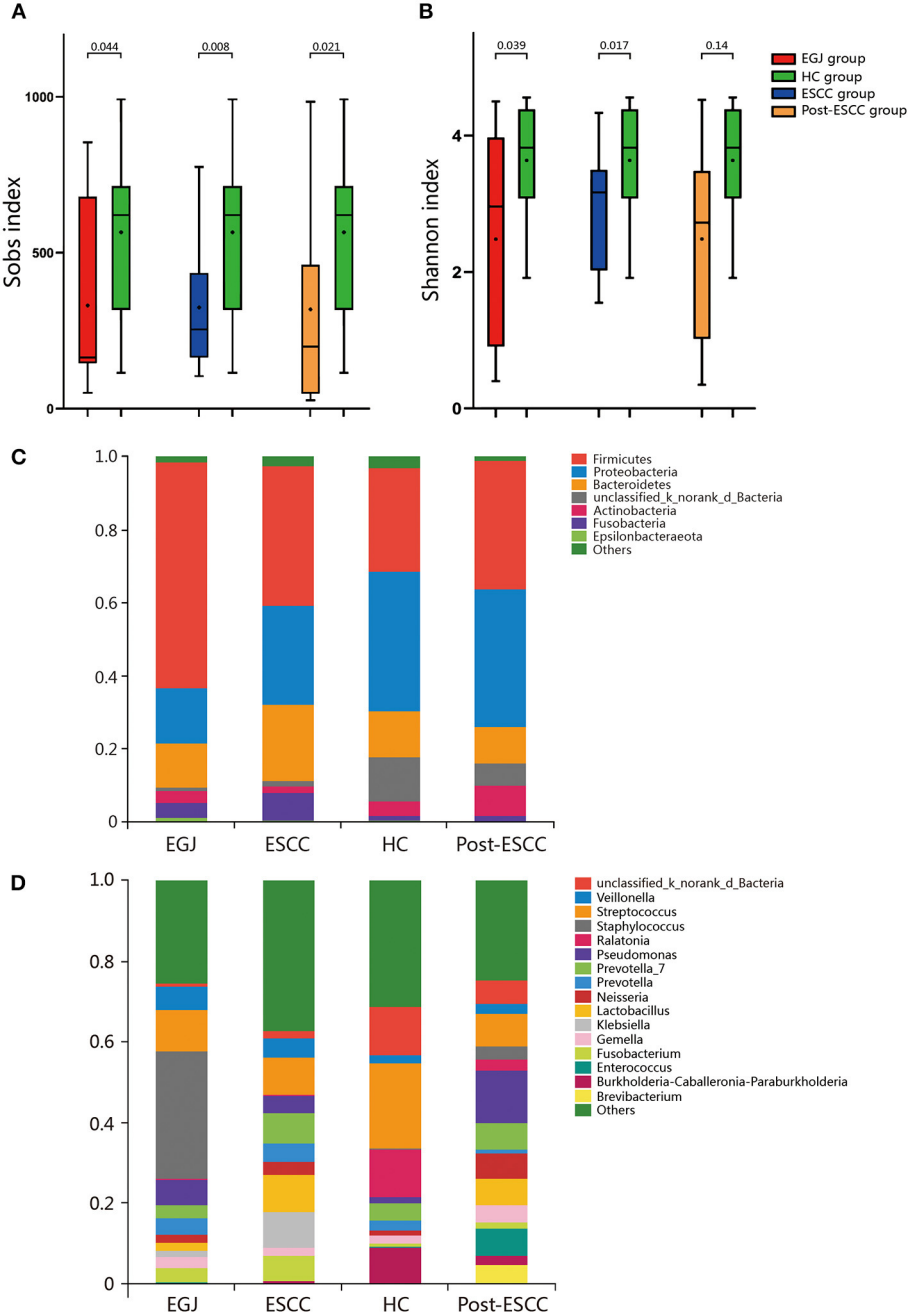
Comparison of α diversity and the relative abundance of taxa in the healthy patients and those of the EGJ, ESCC, and post-ESCC groups. Estimators of community richness [Sobs index, **(A)**] and diversity [Shannon index, **(B)**] in OTU levels. **(C)** Average relative abundance of taxa at the phylum level. **(D)** Average relative abundance of taxa at the genus level. OTU, operational taxonomic unit.

For community coverage, the value of the Good's coverage for all samples was >99% (Figure S1A), indicating that the sequencing results reflected the bacterial composition of the samples. We also evaluated the flat refraction curve, and the results suggested that the quantity of sequencing data was sufficient (Figure S1B). We examined estimators of community richness (Sobs index), diversity, and evenness (Shannon index). Significant differences were observed in the Sobs and Shannon indexes between the ESCC and HC groups (Sobs, 324.59 vs. 565.69, *P* = 0.008; Shannon, 2.95 vs. 3.64, *P* = 0.017), the EGJ and HC groups (Sobs, 331.36 vs. 565.69, *P* = 0.044; Shannon, 2.48 vs. 3.64, *P* = 0.039), and the post-ESCC and HC groups (Sobs, 318.47 vs. 565.69, *P* = 0.021; Shannon, 2.48 vs. 3.64, *P* = 0.014, Figures 1A,B), indicating that microbial diversity was significantly lower in the ESCC, EGJ, and post-ESCC groups than that in the HC group. In addition, no significant differences were observed in Sobs and Shannon indexes between ESCC and EGJ groups, whose α diversity was not significantly different (Figure S1C).

### Microbial Differences Between Tumor and Healthy Tissues

**Figure 2:**
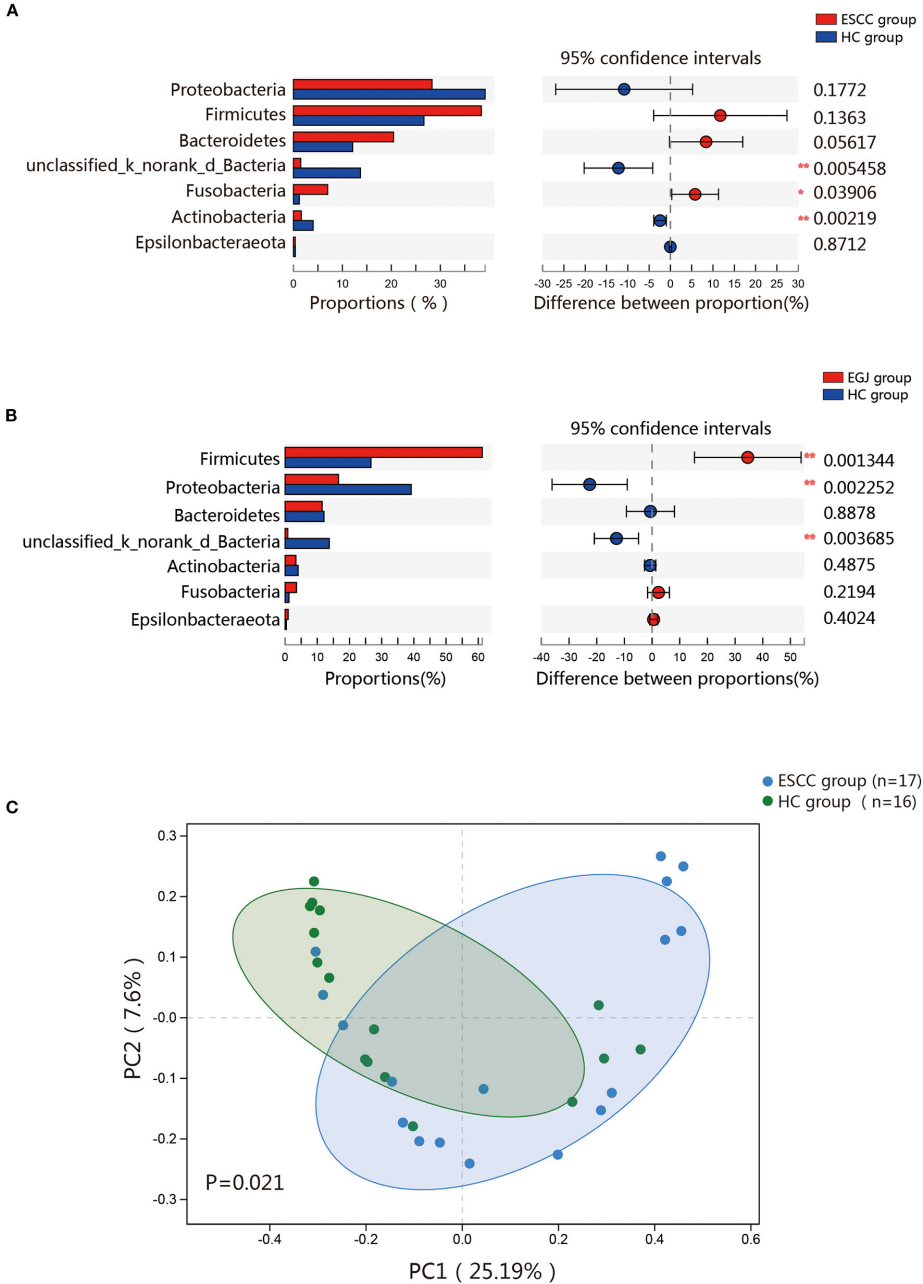
Composition of the esophageal microbiomes of healthy patients and those with ESCC, and β diversity for the EGJ, ESCC, and HC groups. **(A)** Significant differences were observed between the microbiomes of the ESCC and HC groups. **(B)** Significant differences were observed between the microbiomes of the EGJ and HC groups. **(C)** PCoA based on unweighted UniFrac distances between the ESCC and HC groups. *P*-values were calculated by analysis of similarities (ANOSIM) *, 0.01 < *p* < 0.05; **, *p* < 0.01.

We analyzed the microbiomes of the ESCC, EGJ, and HC groups. *Firmicutes* was the predominant phylum in ESCC and EGJ tumor specimens, whereas *Proteobacteria* was the predominant phylum in healthy specimens (Figure 1C). We also examined the microbial community at the genus level. ESCC specimens were rich in *Streptococcus, Lactobacillus, Prevotella*, and *Fusobacterium*, while EGJ specimens were abundant in *Streptococcus, Staphylococcus*, and *Pseudomonas*. By contrast, HC specimens were rich in *Streptococcus, Ralstonia*, and *Burkholderia–Caballeronia–Paraburkholderia* (Figure 1D). We carried out tests of significance between tumor and healthy specimens. The relative abundance of *Fusobacteria* was higher (7.01 vs. 1.12%, *P* = 0.039) and that of *Actinobacteria* was lower (1.61 vs. 4.04%, *P* = 0.002) in the ESCC group than in the HC group (Figure 2A), while the EGJ group showed an increased abundance of *Firmicutes* (61.24 vs. 26.66%, *P* = 0.001) but a decreased abundance of *Proteobacteria* compared with the HC group (16.59 vs. 39.15%, *P* = 0.002, Figure 2B). A difference in the relative abundance of only one phylum, *Firmicutes*, was observed between the ESCC and EGJ groups (38.38 vs. 61.24%, *P* = 0.025, Figure S2A). Interestingly, we identified an unclassified bacterium whose relative abundance was higher in the HC group than in the ESCC and EGJ groups (13.69 vs. 1.50%, *P* = 0.005; 13.69 vs. 0.82%, *P* = 0.003, Figures 2A,B). Compared with the HC group, the relative proportion of *Pseudomonas* increased (5.33 vs. 1.46%, *P* = 0.003) and those of *Ralstonia* and *Burkholderia–Caballeronia–Paraburkholderia* decreased (0.21 vs. 11.38%, *P* < 0.001; 0.77 vs. 9.28%, *P* < 0.001, Table S1) in the ESCC group.

**Figure 3:**
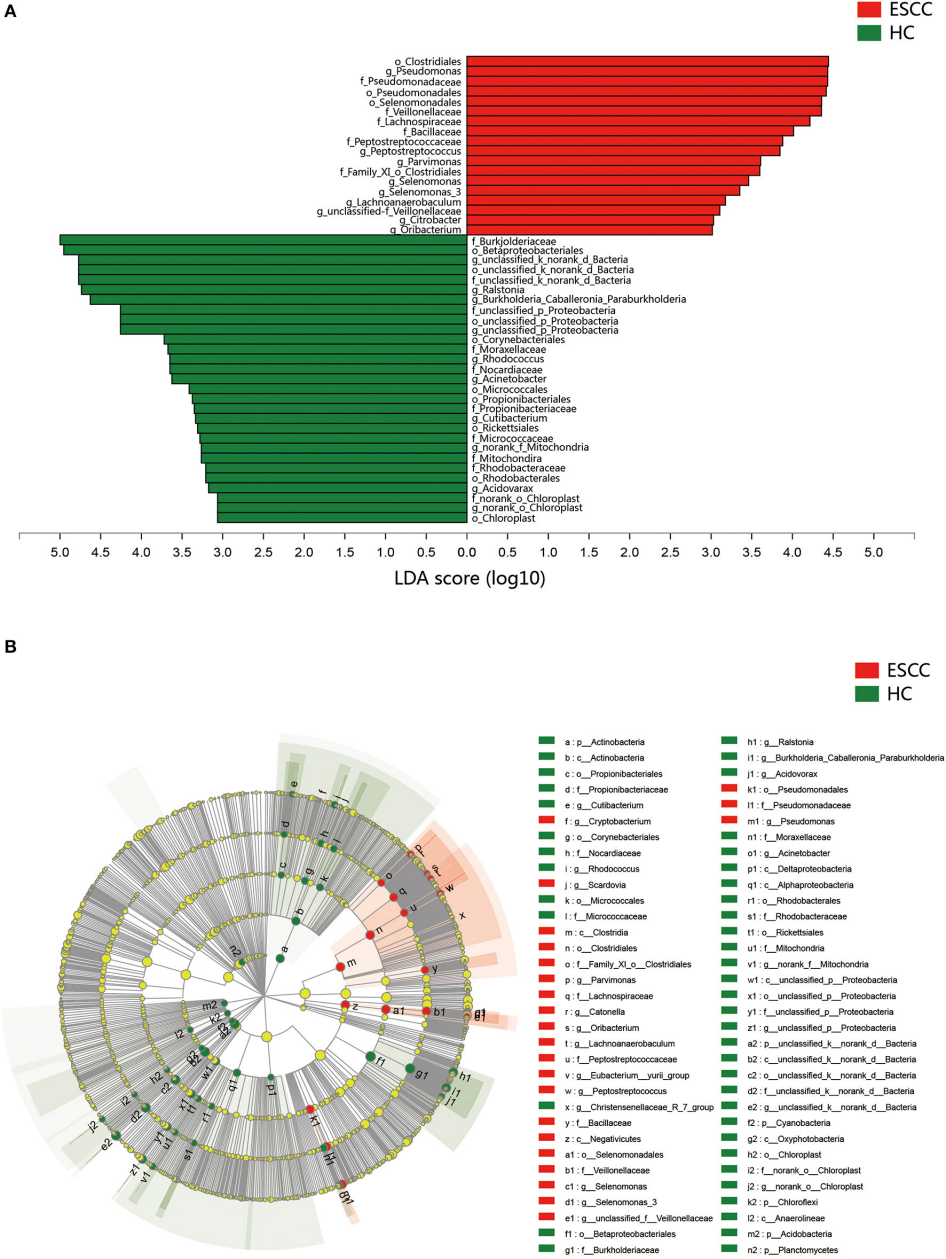
Results of linear discriminant analysis (LDA) and effect size measurements (LEfSe) between the ESCC and HC groups. **(A)** Bar plot shows taxa with LDA score >3.0 from the order to the genus level. **(B)** LEfSe analysis shows the most abundant taxa from the phylum to the genus level between the ESCC and HC groups.

To further evaluate differences in the esophageal microbiome, we examined the β diversity and represented the results of principal coordinate analysis (PCoA) as a plot. The results revealed significant differences between the esophageal microbiomes of the ESCC and HC groups (Figure 2C, Figure S2B). We also examined the LDA coupled with LEfSe. We found that *Clostridiales, Pseudomonas*, and *Selenomonadales* were the key taxa contributing to the changes in the microbiome of ESCC patients, whereas *Burkholderiaceae, Ralstonia*, and *Burkholderia–Caballeronia–Paraburkholderia* were the key taxa contributing to the changes in the microbiome of HC patients (Figures 3A,B). The results of LDA coupled with LEfSe between the EGJ and HC groups are also presented (Figures S2C, S3A).

### Differences Between the Microbial Communities of ESCC and Post-ESCC Patients

**Figure 4:**
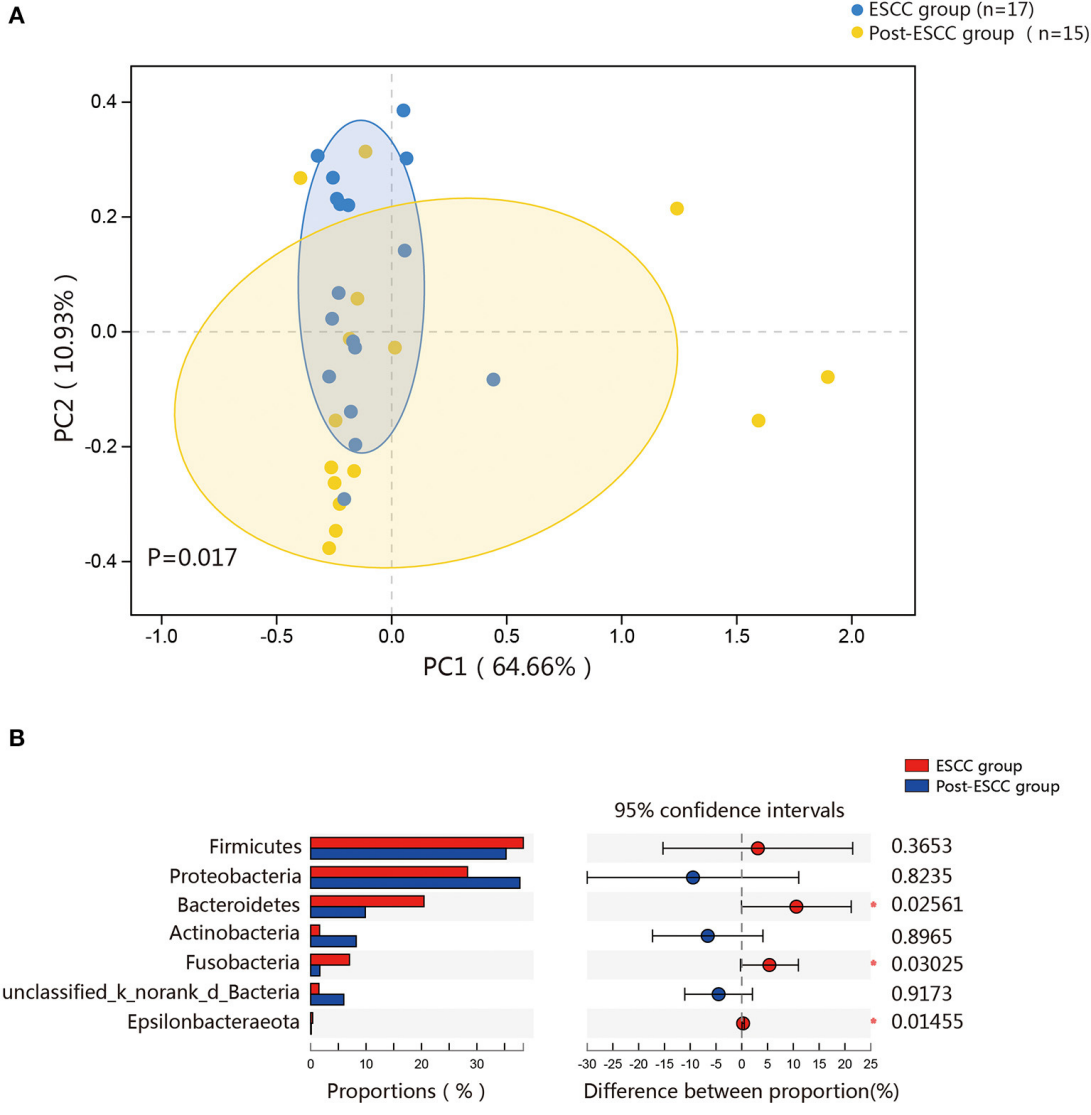
Composition of the esophageal microbiomes and β diversity of the ESCC and post-ESCC groups. **(A)** PCoA based on weighted UniFrac distances between the ESCC and post-ESCC groups. *P*-values were calculated by analysis of similarities (ANOSIM). **(B)** Significant differences were observed between the microbiomes of the ESCC and post-ESCC groups. *P < 0.05.

**Figure 5:**
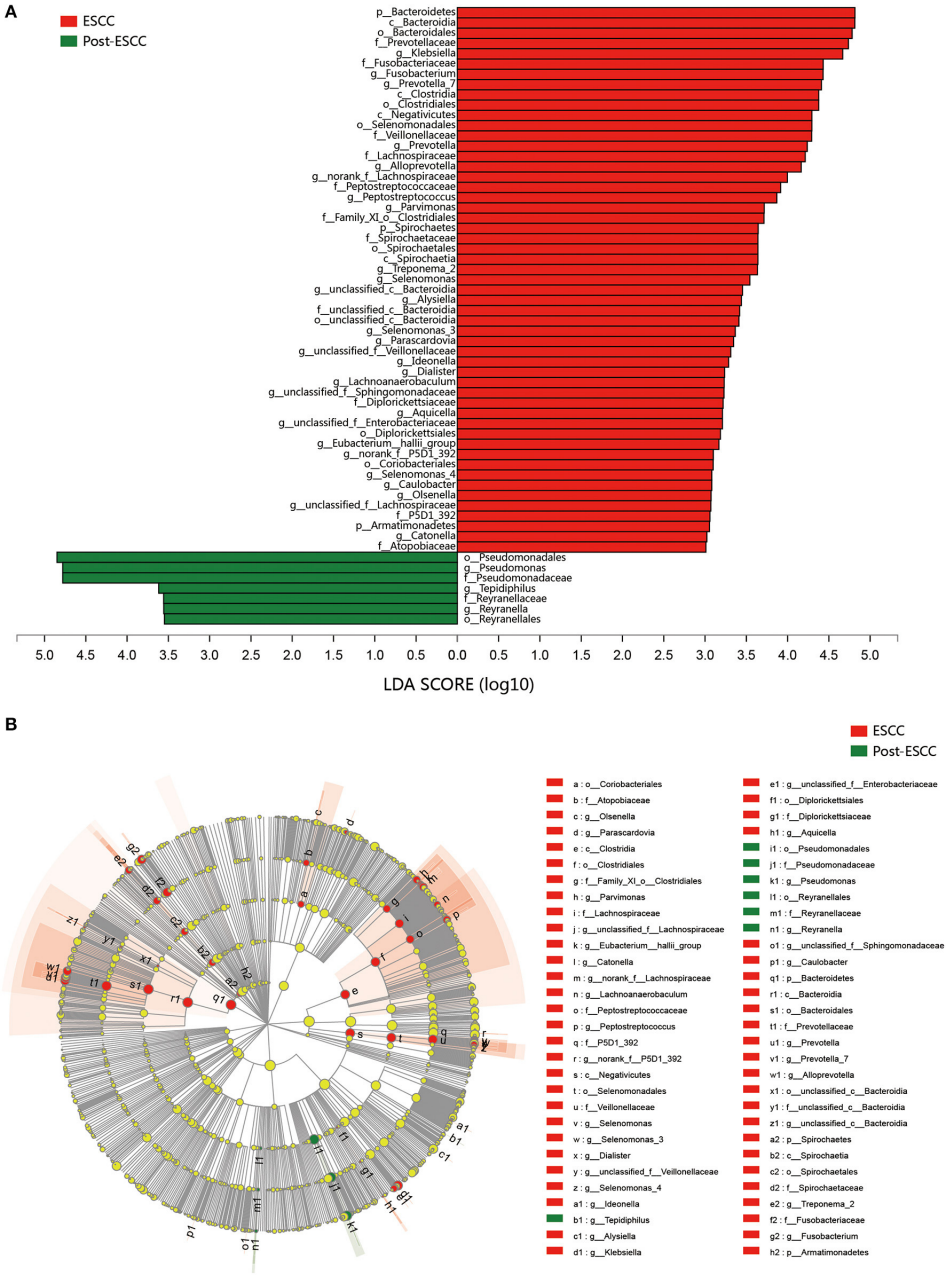
Results of linear discriminant analysis (LDA) and effect size measurements (LEfSe) between the ESCC and HC groups from the phylum to the genus level. **(A)** Bar plot shows results of taxa with LDA score >3.0. **(B)** LEfSe analysis shows the most differentially abundant taxa between the ESCC and post-ESCC groups.

To examine microbial differences caused by esophagectomy, we examined the α and β diversities of the ESCC and post-ESCC groups. There are no significant differences in their Sobs and Shannon indexes (Figure S3B), indicating that the community richness, diversity, and evenness were similar between the two groups. However, the results of PCoA demonstrated that the microbial composition was significantly different (Figure 4A). We found significant differences in the proportions of *Bacteroidetes* (20.45 vs. 9.86%, *P* = 0.026) and *Fusobacteria* (7.01 vs. 1.66%, *P* = 0.030) at the phylum level (Figure 4B) and in the proportions of *Pseudomonas* (5.33 vs. 13.83%, *P* = 0.008), *Fusobacterium* (5.77 vs. 1.25%, *P* = 0.045), and *Prevotella* (4.42 vs. 1.02%, *P* = 0.001) at the genus level between the ESCC and post-ESCC groups (Table S2). The β diversity results suggested that the microbial community of the post-ESCC group was similar to that of the HC group (Figure S4). The results of LDA and LEfSe confirmed that *Bacteroidetes* and *Pseudomonas* were the key taxa contributing to the changes in the microbiome of the ESCC and post-ESCC groups (Figures 5A,B).

### Differences in Microbial Communities Lead to Differences Between the Metabolic Pathways in ESCC and HC Patients

**Figure 6:**
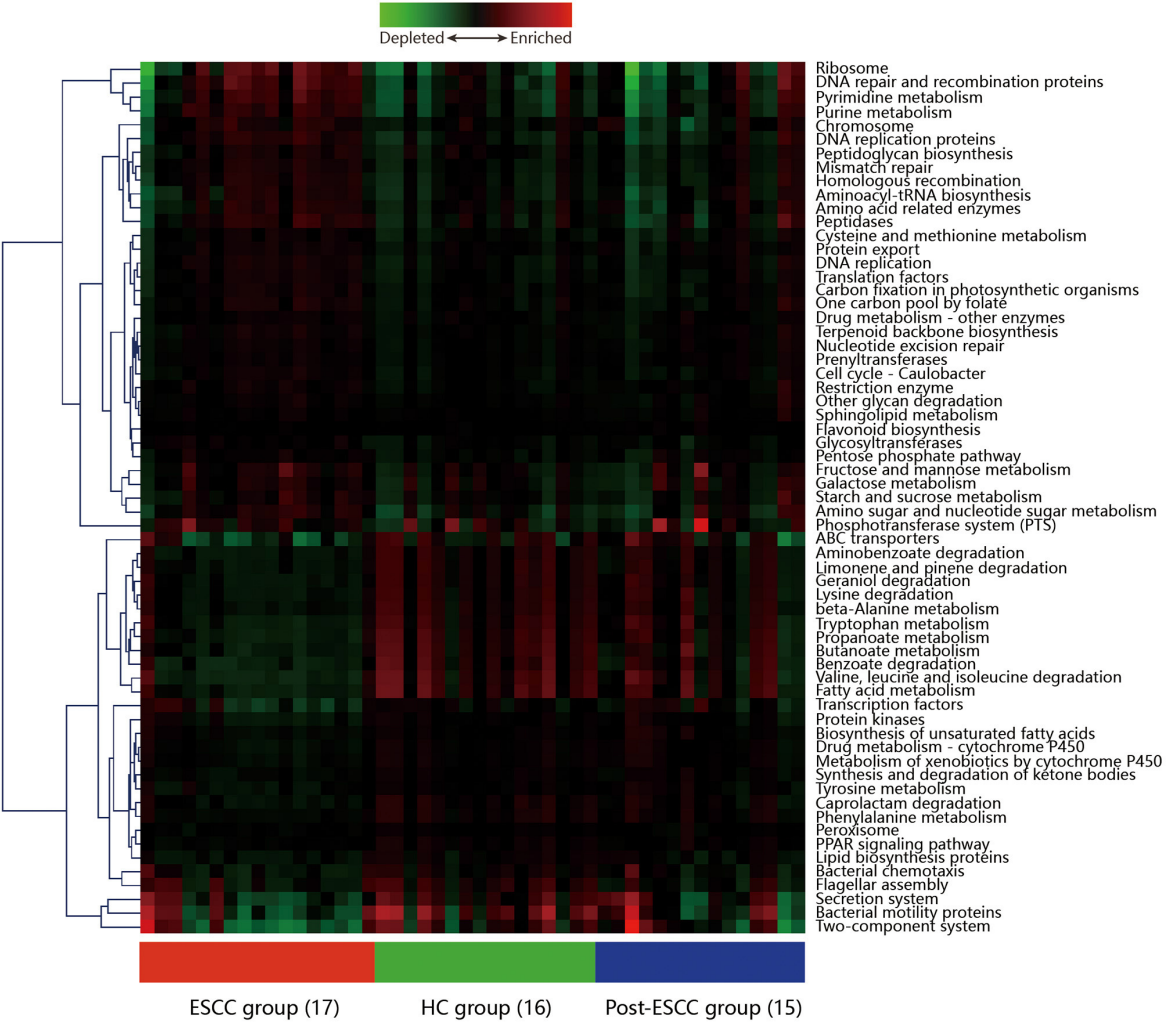
Hierarchical clustering analysis to identify different metabolic pathways. Heatmap of PICRUSt analysis demonstrating the enrichment or loss of metabolic pathways in the ESCC, HC, and post-ESCC groups.

Microbial imbalances can induce systemic metabolic alterations (Nieuwdorp et al., 2014) and vice versa (Cani, 2017). We carried out PICRUSt to predict the metagenomes from the 16S data, and these were used to identify the KEGG pathways involved in ESCC, HC, and post-ESCC specimens. The results are presented as a heatmap. We found that the HC group was enriched in pathways related to the metabolism of fatty acids, short-chain fatty acids (SCFAs), butanoate, propanoate, tryptophan, and beta-alanine, as well as in pathways related to the degradation of benzoate, lysine, geraniol, aminobenzoate, limonene, and pinene. The ESCC group was enriched in pathways related to metabolism of cysteine, methionine, fructose, galactose, and starch, as well as in pathways related to DNA repair and recombination, protein translation, chromosomal dynamics, and peptidase activity (Figure 6). The PICRUSt taxonomic functional relationships suggested that the microbial composition determined which metabolic pathways were involved. Interestingly, the pathways associated with the post-ESCC group were moderately similar to those of the HC group (Figure 6).

## Discussion

Microbes colonize the esophageal mucosa, and increasing evidence suggests that the esophageal microbiome plays important roles in esophageal diseases. Most studies on the esophageal microbiome and esophageal diseases address BE and EAC, but there are few studies concerning ESCC. This is the first study to profile the esophageal microbiome in cancer patients before and after esophagectomy and to predict systemic metabolic alterations. We found the microbial richness and diversity (Sobs and Shannon indexes) to be significantly lower in esophageal cancer and postoperative patients than in healthy controls, which was consistent with the results of a previous study (Castaño-Rodríguez et al., 2017). The α diversity and β diversity were not statistically different between the ESCC and EGJ groups, which is possibly because that the tumor microenvironment creates a niche for similar microbial communities to colonize and thrive even in unique tissue types (Shao et al., 2019). *Proteobacteria* and *Streptococcus* were the predominant taxa in normal esophagus, which was in accordance with earlier studies. In addition, we detected *Ralstonia* and *Burkholderia–Caballeronia–Paraburkholderia*, which were members of the *Betaproteobacteria* family, in healthy controls. *Ralstonia* was the most abundant genus in normal breast tissue and gastric cancer (Tseng et al., 2016; Costantini et al., 2018). Our results showed that *Ralstonia* comprised the microbiome of the normal esophagus, and further studies are needed to verify these results.

The esophagus of patients with ESCC was enriched in *Streptococcus, Prevotella, Fusobacterium, Veillonella*, and *Lactobacillus*, which is in conformity with a previous study (Shao et al., 2019). In Japanese patients with esophageal cancer, *Fusobacterium nucleatum* was enriched in ESCC tissue compared with matched normal esophagus tissue, and is quantity was negatively correlated with survival (Yamamura et al., 2016, 2019). *F. nucleatum* has potential prognostic value for ESCC and had been detected in colon cancer. Other studies have reported a robust association between *F. nucleatum* and colorectal cancer (Castellarin et al., 2012; Kostic et al., 2013; Rubinstein et al., 2013; Yu et al., 2017). The *F. nucleatum* presented in these tissues had been verified by isolating *F. nucleatum* strains directly from biopsy samples and from patient-derived xenografts passaged in mice (Castellarin et al., 2012; Bullman et al., 2017). *F. nucleatum* can induce expression of the pro-inflammatory cytokines in epithelial cells, including IL-6 and IL-8 (Ahn et al., 2017), which can contribute to the dynamic cross-talk between tumor cells and cancer-associated fibroblasts (CAF) in the TME for ESCC (Karakasheva et al., 2018). *F. nucleatum* infection can induce expression of the antimicrobial peptide β-defensin 2 and high-mobility group box 1 protein (HMGB1) (Bui et al., 2016), which can result in increased proliferation and modulated autophagy in ESCC cell lines (Di et al., 2019). In the ESCC group, the relative abundances of Clostridiales and Pseudomonas also significantly increased compared to the HC group. *Clostridiales* are obligate anaerobes that thrive in hypoxic environments such as those created by tumors. A previous study reported that *Clostridiales* in the gastric corpus could contribute to esophageal squamous dysplasia (Nasrollahzadeh et al., 2015). Transplanting *Clostridium* to germ-free nutrition-deficient mice can increase the acylcarnitine level in the gut and decrease protein synthesis and amino acid oxidation in the liver (Blanton et al., 2016), thereby promoting the development of cancer. *Clostridiumalos difficile*, as a member of the *Clostridiales* class, also plays a pivotal role in regulating *Clostridium difficile* infections (CDI) (Farowski et al., 2019). The Gram-negative *Pseudomonas* is over-represented in cases of oral squamous cell carcinoma (OSCC) (Perera et al., 2018), a kind of tumor that is similar to ESCC. *Pseudomonas* can induce Toll-like receptors to activate cytokines, chemokines, and COX-2 and recruit cells of the innate and adaptive immune system (Markou and Apidianakis, 2014). Infection with *Pseudomonas* could activate the c-Jun N-terminal kinase (JNK) pathway to induce enterocyte apoptosis, and intestinal stem cell proliferation (Apidianakis et al., 2009), intestinal innate immune responses, and stem cells may initiate the development of tumors and metastasis (Schwitalla et al., 2013). In addition, no significant change was observed in the relative abundance of *Porphyromonas gingivalis* in our study, which was evidenced in esophageal cancer and dysplasia tissues but was rarely found in non-cancerous and normal tissues (Gao et al., 2016).

We also noticed differences in the esophageal microbiome after surgery. At the genus level, the abundance of *Fusobacterium* and *Prevotella* decreased after surgery. In another study, the relative proportions of *Firmicutes* and *Bacteroidetes* increased, while those of *Proteobacteria* and *Actinobacteria* decreased after gastrectomy (Tseng et al., 2016). *Bacteroidetes* are Gram-negative anaerobes and micro-aerophiles that reside in the diseased esophagus (Yang et al., 2009). Compared to the HC group, *Lactobacillus* significantly increased in the post-ESCC group, which was associated with the acidic microenvironment caused by the increased gastric acid reflux after esophagectomy. Another study also indicated that a low pH is needed for the proliferation of *Lactobacillus* (Elliott et al., 2017). The gastric microenvironment is populated by microbial communities mainly comprised of the Lactobacillus, Streptococcus, and Propionibacterium genera (Nardone et al., 2017).

The results of PICRUSt showed that the pathways related to the metabolism of cysteine and methionine, monosaccharides, starch were upregulated and the metabolic levels of fatty acids, short-chain fatty acids (SCFAs), tryptophan, and beta-alanine were low in the ESCC group. Meanwhile, pathways related to other cellular functions, including DNA repair and recombination, protein translation, chromosomal dynamics, and peptidase activity were also altered. Methionine uptake and breakdown associate with various cellular processes such as methylation reactions, polyamine synthesis, and redox maintenance. SCFAs are produced by colonic microbial fermentation of undigested or partially digested dietary fibers and have broad effects on host immune system development and function, which can enhance epithelial barrier function and immune tolerance (Rooks and Garrett, 2016). Tryptophan has been shown to play a crucial role in the balance between intestinal immune tolerance and gut microbiota maintenance (Lee and Lee, 2010). The metabolic heterogeneity between the ESCC and HC groups was essential for host immune function and tumorigenesis. After radical esophagectomy, the microbial metabolism became similar to that of healthy control, suggesting that tumors can alter the microbial microenvironment and metabolic profile. The microbial composition of the esophagus may tend to become similar to normal status after surgery. It is suggested that the microbiota composition and metabolism profile are associated with the physiological or pathological status of the esophagus and that the emergence of some microbes, which dominated in the tumor patients, may be predictors of the recurrence of the tumor.

Of course, there were several limitations to this study. First, the sample size of each group was limited, and larger studies should be performed to validate our findings. Second, the microbiome that we studied was likely to be contaminated by saliva during endoscopic sampling. Finally, we only examined the microbiome at 9–12 months after surgery. Additional time points may better describe the changes in the microbial microenvironment for postoperative cases. In addition, investigations of the biological mechanisms of the microbiota are needed to elucidate the association between complex microbial environments and esophageal cancer.

In conclusion, we compared the microbial composition of esophagus in different statuses and found some microbiota associated with the tumorigenesis. We also discovered the microbial metabolism of postoperative esophagus to be similar to that of healthy tissue. Monitoring of the esophagus microbiota may be an essential method for predicting the recurrence of tumor.

## Data Availability Statement

All sequencing data associated with this study have been uploaded to the NCBI SRA database (SRA accession: PRJNA628659).

## Ethics Statement

The studies involving human participants were reviewed and approved by the Institutional Review Board of the Renmin Hospital of Wuhan University. The patients/participants provided their written informed consent to participate in this study.

## Author Contributions

DL contributed to study concept and design, data collection, analysis, and interpretation, and review of the manuscript. RH was responsible for the data collection, analysis, and interpretation and drafting of the manuscript. TF worked on the interpretation and review of the manuscript. GH helped with data collection, interpretation, and review of the manuscript. WM, LC, LZ, WJ, WW, ZL, and HF was involved in data collection and review of the manuscript. QG contributed to study concept and design, data collection, analysis, and interpretation, and critical review of the manuscript. All authors contributed to the article and approved the submitted version.

## Conflict of Interest

The authors declare that the research was conducted in the absence of any commercial or financial relationships that could be construed as a potential conflict of interest.
