# Supplementary material for: Characterization of the Esophageal Microbiota and Prediction of the Metabolic Pathways Involved in Esophageal Cancer
Source: Front Cell Infect Microbiol. 2020 Jun 26;10:268. doi: 10.3389/fcimb.2020.00268 (PMC7333312; doi:10.3389/fcimb.2020.00268)
Supplement: Supplementary file 1 [file Data_Sheet_1.ZIP › Supplementary Material.docx]

Supplementary Material

# Supplementary Figures and Tables

## Supplementary Figures


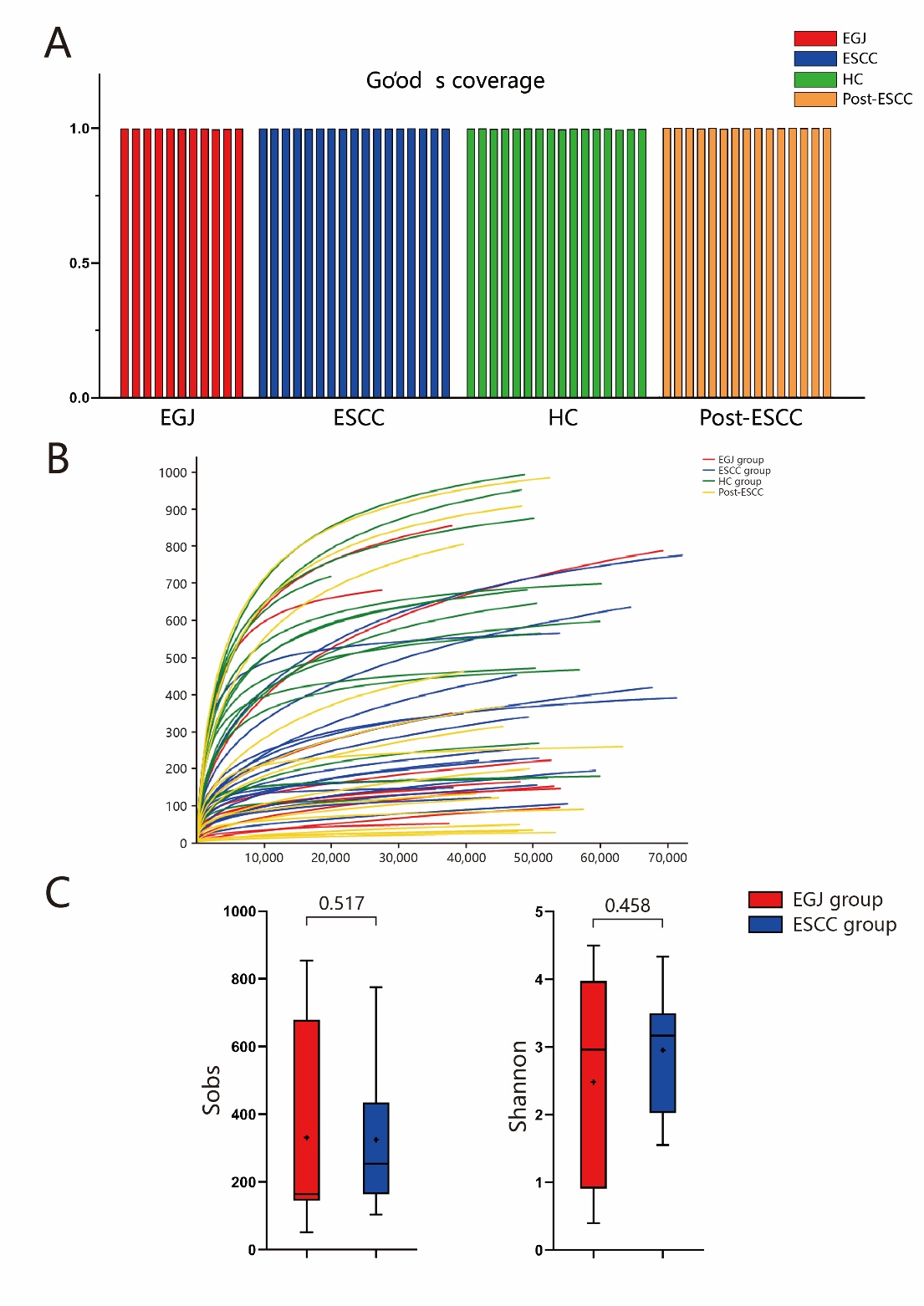


**Figure S1.** (A) The microbial coverage based on OTU. (B) Refraction curve on OUT level. (C) Estimators of community richness (Sobs index) and diversity (Shannon index) on OUT level.

**
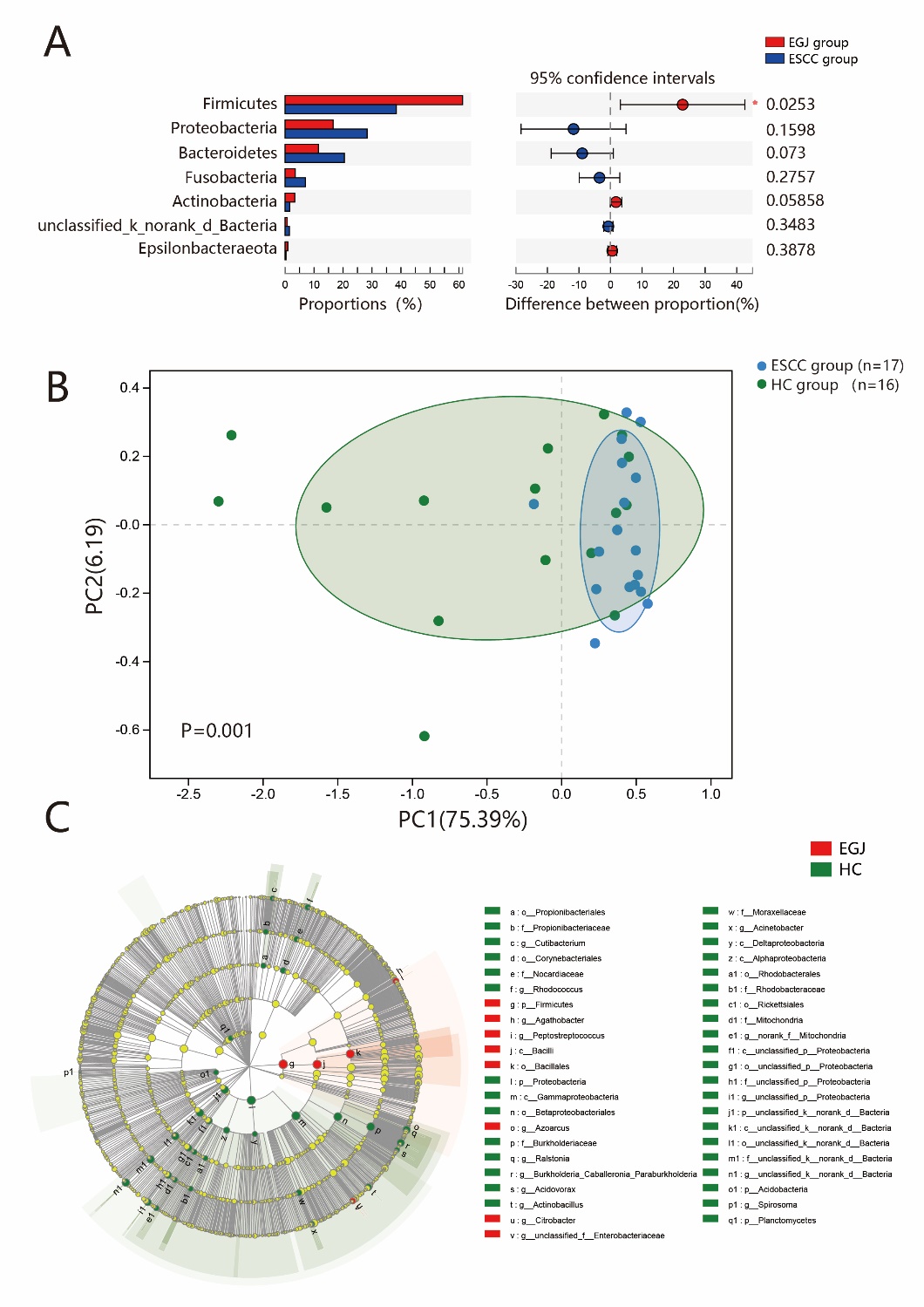
**

**Figure S2.** (A) The microbial differences on phylum level between the EGJ and ESCC groups. (B) PCoA based on weighted UniFrac distances between ESCC and HC groups. *P*-values were calculated by the analysis of similarities (ANOSIM), (C) LEfSe analysis shows the most abundant taxa from the phylum to the genus level between EGJ and HC groups. *, 0.01 < *p* < 0.05; **, *p* < 0.01.


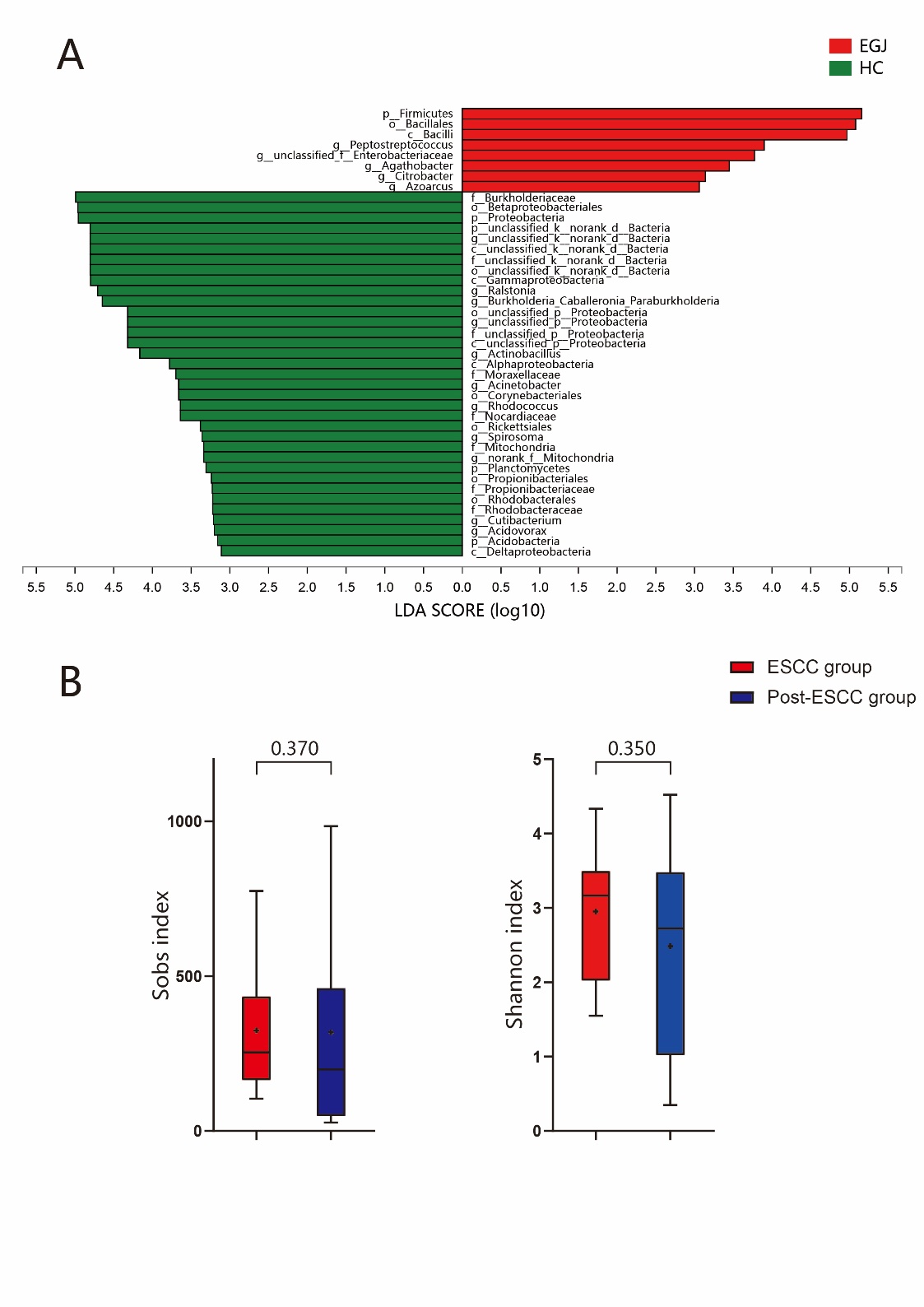


**Figure S3.** (A) Bar plot shows taxa with an LDA score >3.0 from the phylum to the genus level. (B) The α diversity based on OTU (Sobs, Shannon) between ESCC and Post-ESCC groups.


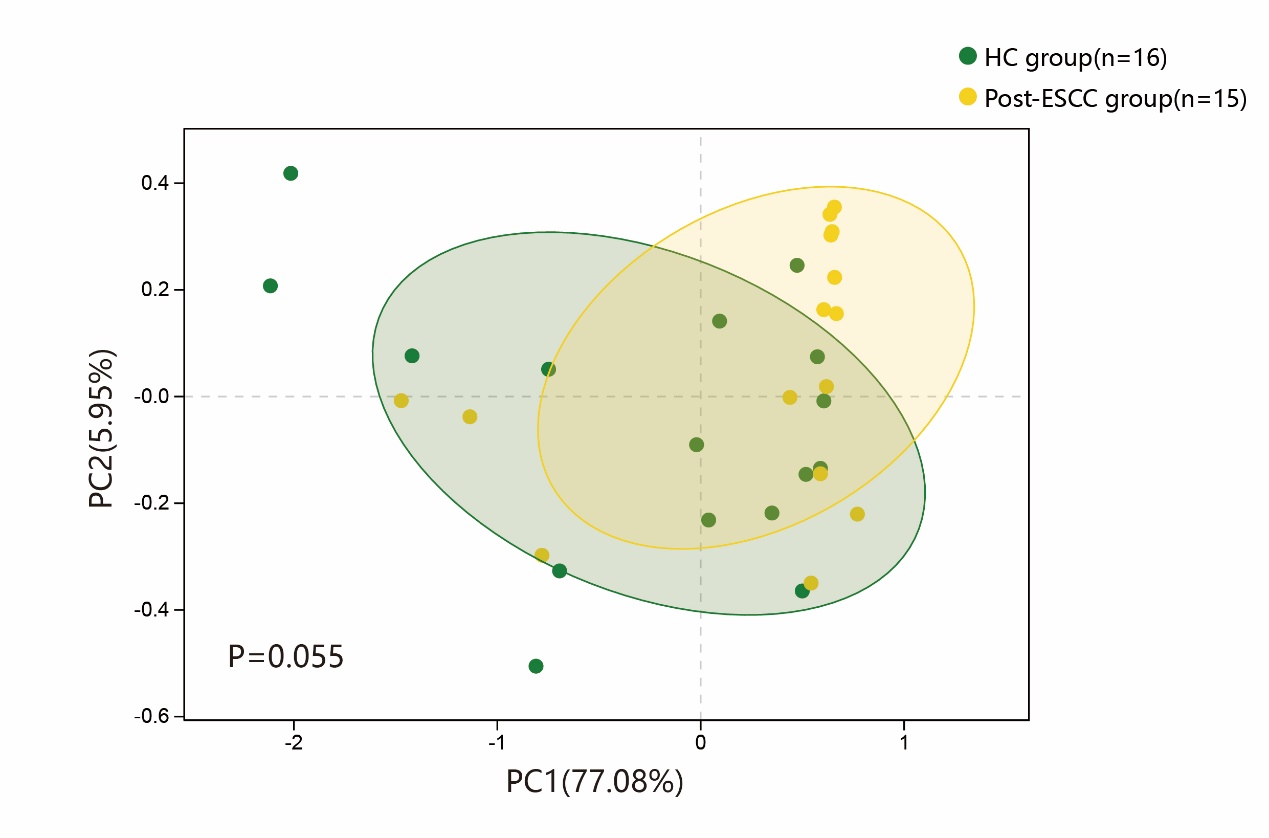


**Figure S4.** PCoA based on weighted UniFrac distances between HC and Post-ESCC groups, The P-value was calculated by the analysis of similarities (ANOSIM).

**Supplementary Tables**

There were two supplementary tables. Due to the limited contents of the form, it will be uploaded separately.
